# Transactions of the Entomological Society of London

**Published:** 1841-07

**Authors:** 


Art. III.—*Transactions of the Entomological Society of London.* Vol. II. Parts III. & IV.—*London*, 1839-40. 4to.

We are more particularly induced to notice this work, from the circumstance that it contains some interesting communications on the occasional presence of insects or their larvæ as parasites in the human body. We refer more especially to two papers, the one by the Rev. Mr. Jenyns in the third part of this volume, and the other by the Rev. F. W. Hope in the fourth part.

In Mr. Jenyns's paper are detailed the circumstances of a well-authenticated case of the existence of an immense number of larvæ of a dipterous insect, supposed to be the *anthomyia canicularis,* in the alimentary canal of a patient, under the care of Dr. Haviland of Cambridge, in the autumn of 1836. This patient was a clergyman, seventy years of age, and the symptoms of which he complained, previously to the first expulsion of the larvæ from the bowels, as stated by Dr. Haviland were :

"General weakness, loss of appetite, and a disagreeable sensation about the epigastrium, which he (the patient) described as a tremulous motion. These symptoms commenced in the spring of 1836, and it was not till the summer and autumn of that year that the larvæ were observed in the motions. They then passed off in very large quantities on different occasions, the discharge continuing for several months. According to the patient's own statement, the chamber vessel was sometimes half full of these animals, at other times they were mixed with the stools. He thinks that altogether the quantity evacuated must have amounted to several quarts. The larvæ were all nearly of equal size, and, when first passed, quite alive, moving with great activity. The patient is not aware of having voided anything of the kind before." (pp. 152-3.)

Mr. Jenyns remarks that, after the discharge had ceased, the patient's health was improved, but not completely reestablished. He observes also that these larvæ appear to be similar to those in a case recorded by Dr. Bateman,* that they were most likely introduced into the stomach in the state of ova, and had remained there, and in the intestines, until expelled by stool, a supposition highly probable from the fact that, according to Degeer, the *anthomyia* usually reside in the larva state in the ordure of privies, cesspools, and other filthy places, for which their organs of respiration, minutely described by Mr. Jenyns, peculiarly adapt them.

In Mr. Hope's paper are collected together a great number of cases recorded by many of the most distinguished naturalists and physiologists, in which insects or their larvæ have been found in the human body. These are arranged in the form of tables, which appeared in an imperfect state, some time since in the Medical Gazette. The disease occasioned by insects or their larvæ was some years ago designated by the distinguished naturalist the Rev. Mr. Kirby, *scholechiasis.* Mr. Hope proposes to retain this term for the disease only when occasioned by the larvæ of *lepidopterous* insects, and to use that of *canthariasis* when the disease results from the presence of the larva or perfect individuals of coleoptera; and *myacis* when induced by the larva or perfect state of diptera. We certainly do not perceive the advantage of employing these different terms to indicate the same disease, because

* Edinburgh Medical and Surgical Journal, vol. vii. p. 41.

occasioned by a different *species* of insects, since the symptoms of the existence of the parasite will vary, perhaps not so much in consequence of the different kind of animal as of the different part of the body in which it is occasioning irritation, and inducing the disease by its presence. To us it appears more correct to retain the term originally proposed as a kind of *generic* distinction, and to use those now proposed by Mr. Hope, or others that may appear more suitable, as *specific* terms, in describing the *immediate* object occasioning the disease, especially as the symptoms occasioned by all kinds of insects are very similar, and as the species cannot be known until some of the parasites have actually been expelled from the body.

On examining these tables, we have been struck with the circumstance that more than one half of the whole of the cases recorded have been occasioned by the larva of *dipterous* insects, a circumstance worthy of remark, from the fact that these larvæ, from their structure and habits, are best adapted to reside in closed moist localities, and consequently are those most likely to exist as parasites in the body. Much uncertainty prevails as to the manner in which these animals are usually introduced into the cavities of the body, and the more so from their having been found, especially the dipterous larvæ, in organs in which it is hardly possible to conceive they could have obtained an entrance. On inspecting Mr. Hope's tables it will be seen that they have been found in the frontal sinus, in the nose, the bladder and urinary passages, the antrum maxillare, the stomach, and the intestines. Those which have been found in the antrum and frontal sinus have been almost entirely larvæ of the *œstri* and *muscæ*. On the reading of Mr. Hope's paper, we observe from the Proceedings of the Society, that Mr. Bracy Clark expressed his doubts on the occurrence of œstri in the alimentary canal or frontal sinus of man, and also of there being any species of œstrus peculiar to the human subject, as supposed, and named in the two cases of inflammatory tumour described by Mr. Howship, in the Proceedings of the Royal Society ;* the larvæ in those cases being regarded by him as those of the *œstrus bovis*. At a subsequent meeting, however, Mr. Newport related a case which had been communicated to him by Dr. Carter, of a female out-patient of the Canterbury hospital, who after suffering for a long period with severe anomalous pains in the region of the frontal sinus, at length discharged from the nostrils one perfect living larva of an œstrus, and subsequently portions of two others; after which the pains in the head ceased, and the patient recovered. He mentioned also that a species of *geophilus* had once been voided from the stomach of a patient under his own care. Professor Owen also detailed a case, and produced specimens of the larvæ of a dipterous insect, apparently similar to those in Mr. Jenyns's case, which had been sent to him by a medical man in the country, and which had actually been passed from the urethra by the patient, an aged gentleman, in the presence of the medical attendant. Some of these larvæ had been kept alive in urine for two or three days, but Professor Owen was unable to rear the perfect insect.

Mr. Hope suggests that these insects are conveyed into the stomach in the egg, or the early larva state with the food; but this will not explain how they are admitted into such localities as the antrum and frontal

* Vol. iii. p. 181.

sinus, and above all the bladder. That they are taken into the stomach with the food, in the state of ova, is, we think, highly probable; and that being developed in a locality the temperature of which is almost always the same, they not merely suffer no inconvenience from its great amount, but even thrive in that in which, under other circumstances, they would have perished, seems to explain the mystery of their continued growth and residence in the body. We consider the subject of their introduction into such localities as the urinary bladder as meriting the particular attention of the profession. That some animals of this class are occasionally swallowed in the unfiltered water drunk, more especially by the poorer classes of this metropolis, we have not the slightest doubt, since we have ourselves found the water supplied from the reservoirs, in the summer, swarming with living beings; not merely the common infusorial animals which exist in almost all waters, but with living and, in many instances, perfect insects. We may particularize several species we have noticed, among which the little crustaceous animal *cypris conchacea* is exceedingly common, the *notonecta*, or water boatmen, and the *asellus aquaticus* and some *planariæ*. When it is remembered that these may often be swallowed in the water drunk during summer by the poor, the presence of these creatures becomes a matter of consideration in regard to the public health, and leads us to suspect that many complaints, otherwise anomalous in symptoms, may perhaps, on close examination be traced to this cause. That their presence has sometimes been attended by fatal results is clearly shown in Mr. Hope's tables, which we regard as meriting the attention of the profession, and which we trust will incite its members to a closer investigation of this important subject.

